# SARS-CoV-2 surveillance in untreated wastewater: detection of viral RNA in a low-resource community in Buenos Aires, Argentina

**DOI:** 10.26633/RPSP.2021.137

**Published:** 2021-10-18

**Authors:** Néstor Gabriel Iglesias, Leopoldo Germán Gebhard, Juan Manuel Carballeda, Ignacio Aiello, Emiliano Recalde, Gabriel Terny, Silvina Ambrosolio, Gabriela L’Arco, Jonatan Konfino, Juan Ignacio Brardinelli

**Affiliations:** 1 Universidad Nacional de Quilmes Buenos Aires Argentina Universidad Nacional de Quilmes, Buenos Aires, Argentina.; 2 Consejo Nacional de Investigaciones Científicas y Técnicas de Argentina (CONICET) Buenos Aires Argentina Consejo Nacional de Investigaciones Científicas y Técnicas de Argentina (CONICET) Buenos Aires, Argentina.; 3 Organismo Provincial para el Desarrollo Sostenible Buenos Aires Argentina Organismo Provincial para el Desarrollo Sostenible, Buenos Aires, Argentina.; 4 Organismo Provincial de Integración Social y Urbana Buenos Aires Argentina Organismo Provincial de Integración Social y Urbana, Buenos Aires, Argentina.; 5 Municipalidad de Quilmes, Buenos Aires Buenos Aires Argentina Municipalidad de Quilmes, Buenos Aires, Argentina.

**Keywords:** Environmental monitoring, SARS-CoV-2, wastewater, Argentina, Monitoreo del ambiente, SARS-CoV-2, aguas residuales, Argentina, Monitoramento ambiental, SARS-CoV-2, águas residuárias, Argentina

## Abstract

**Objective.:**

To measure SARS-CoV-2 RNA in sewage in a low-resource community in order to determine if it can be considered as an estimator of changes in the prevalence of COVID-19 in the population.

**Methods.:**

In this descriptive observational study we collected samples of surface waters contaminated with sewage and optimized a method of purification of viral RNA using PEG concentration. We determined the amount of genetic material by quantitative real-time PCR using the CDC method for SARS-CoV-2 detection.

**Results.:**

We quantified viral RNA in surface waters contaminated with sewage of a low resource community and determined that temporal trends of SARS-CoV-2 in wastewater samples mirrored trends in COVID-19 active cases.

**Conclusions.:**

Measuring of SARS-CoV-2 RNA in sewage can be applied in low-resource communities without connection to sewers as an estimator of changes in the prevalence of COVID-19.

## RESUMEN

**Objetivo.:**

Medir el ARN del SARS-CoV-2 en las aguas residuales de una comunidad de bajos recursos para determinar si se puede considerar un estimador de cambios en la prevalencia de la COVID-19 en la población.

**Métodos.:**

En este estudio descriptivo de observación se tomaron muestras de las aguas superficiales contaminadas con aguas residuales y se optimizó un método de purificación del ARN viral mediante la concentración de polietilenglicol (PEG). Se determinó la cantidad de material genético por PCR cuantitativa en tiempo real empleando el método de los CDC para la detección del SARS-CoV-2.

**Resultados.:**

Se cuantificó el ARN viral de las aguas superficiales contaminadas con aguas residuales de una comunidad de bajos recursos y se determinó que las tendencias temporales del SARS-CoV-2 en las muestras de aguas residuales reflejaban las tendencias en los casos activos de COVID-19.

**Conclusiones.:**

La medición del ARN del SARS-CoV-2 en aguas residuales puede aplicarse en las comunidades de bajos recursos sin conexión al alcantarillado como un estimador de los cambios en la prevalencia de la COVID-19.

## RESUMO

**Objetivo.:**

Medir o RNA do SARS-CoV-2 nas águas residuais de uma comunidade com poucos recursos, a fim de determinar se pode ser utilizado para estimar mudanças na prevalência de COVID-19 na população.

**Métodos.:**

Neste estudo observacional descritivo, coletamos amostras de águas superficiais contaminadas com dejetos e otimizamos um método de purificação de RNA viral utilizando concentração de PEG. Determinamos a quantidade de material genético por PCR quantitativo em tempo real, usando o método do CDC para detecção de SARS-CoV-2.

**Resultados.:**

Quantificamos o RNA viral em águas superficiais contaminadas com dejetos de uma comunidade com poucos recursos e determinamos que as tendências temporais do SARS-CoV-2 em amostras de águas residuais refletiam tendências de casos ativos de COVID-19.

**Conclusões.:**

A mensuração do RNA do SARS CoV-2 em águas residuais pode ser aplicada em comunidades com poucos recursos, sem ligação com a rede de esgoto, para estimar mudanças na prevalência de COVID-19.

Wastewater-based epidemiology (WBE) provides comprehensive health information at the community level (1). The concept is mainly based on the detection and analysis of chemical and biological compounds in sewage. WBE is an approach used to monitor the presence of pathogens which may pose a public health concern (2). During the current COVID-19 pandemic, sewage surveillance by analyzing SARS-CoV-2 RNA traces in wastewater has been reported in many locations around the world. All these studies have been conducted in populations which have sewer networks and wastewater treatment facilities (3-15). However, no study has been reported with this approach in low-resource settings lacking these facilities. Moreover, the World Health Organization (WHO) in a brief report about the status of environmental surveillance for SARS-CoV-2 indicated that approaches are needed that can be applied in low-resource settings, where a greater proportion of the population is not connected to sewers and instead uses pit toilets or septic tanks. These possibilities include testing surface water contaminated by sewage. On January 10 2020 the WHO published a comprehensive package of guidance documents for countries covering topics related to the management of an outbreak of a new disease, including recommendations about surveillance. On March 11 2020, the WHO declared COVID-19 as a pandemic, and on March 18 the first case was confirmed in the Municipality of Quilmes, Province of Buenos Aires, Argentina and the prevention and control strategy of COVID-19 began to be implemented. On May 25 a COVID-19 outbreak in the Villa Azul neighborhood of Quilmes was confirmed and an outbreak mitigation strategy was implemented in addition to a comprehensive surveillance strategy that also was extended to Villa Itatí community given the geographical and social proximity (16).

In this scenario, a surveillance strategy was developed in Villa Itatí including an active search of eventual new cases (suspected cases) house by house, registry of new COVID-19 cases and wastewater surveillance. Every suspected case under the current epidemiological guidelines was tested with a nasal and oral swab for real time PCR analysis. Once the case was confirmed, isolation measures were recommended for the recovery and avoidance of additional contagiousness and cases (16).

The aim of this study was to determine whether SARS-CoV-2 RNA levels in sewage-contaminated surface waters can be considered a reliable estimator of changes in the prevalence of COVID-19 at the population level in a low-resource community not connected to sewer.

## MATERIAL AND METHODS

**FIGURE 1.**
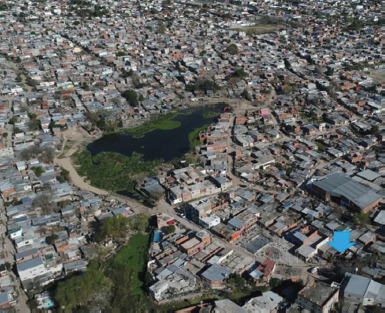
Aerial view of Villa Itatí neighborhood, Quilmes, Buenos Aires, Argentina with the lagoon and the pumping station indicated (arrow). Source: Ariel Romaniuk, Municipality of Quilmes

In this descriptive observational study, raw surface water samples were collected between June 5 and September 7, 2020 at Villa Itatí neighborhood, Municipality of Quilmes, Buenos Aires, Argentina (Figure 1). It is estimated that Villa Itatí has a population of 16 478 people, in an area of 55 hectares delimited by Montevideo, Levalle, Ayacucho and Southeast access streets. The neighborhood has an average of 1.03 households per dwelling with an average of 3.55 people. Of the 4 261 homes in Villa Itatí, 3 966 (93.1%) have potable water from the network and 1 044 (24.5%) are connected to the public sewer network (http://www.estadistica.ec.gba.gov.ar/dpe/index.php/censos).

This community is located around an endorheic urban basin formed by an anthropogenic digging flooded by the groundwater table and the discharges of pluvial and domestic waters (Figure 1) (17). Most households serve their sewage into the waterlogged digging (lagoon) through open drains or indirectly by underground infiltration from pit toilets and cesspools. In order to avoid the overflowing of the lagoon, its content is daily discharged to the urban rainwater network through a pumping system (17). Water samples were taken from the only pumping station of the neighborhood. We collected raw water samples 11 times from June to September at Villa Itatí pumping station. Sampling was performed weekly with some exceptions mainly due to impediments caused by heavy rains.

Composite samples (200 ml each 20 minutes) taking in a 6-hour period were collected from 8 a.m. to 2 p.m. in sterilized glass bottles and kept at 4 °C until analysis. During the sampling, the pumping station was in operation, discharging the contents of the lagoon into the sewage network. RNA purification and viral detection were performed the same day of the sample collection. Before processing, samples of 500 ml were subjected to 90 minutes treatment at 60 °C to ensure biological safety. We optimized an RNA isolation procedure consisting of a PEG_8000_-based precipitation step in order to achieve viral concentration from the raw water samples. Then, a TRIzol (chaotropic agents/organic solvents, Invitrogen) extraction followed by a silica-gel-based RNA purification step were carried out in order to recover the viral RNA free from interfering substances. Viral concentration was carried out from 250 ml of sample adding 20 g of PEG_8000_ and 4.5 g of NaCl and then centrifuging at 12 000 ×g during 1 hour at 4 °C. The pellet was suspended in 1 ml of TRIzol and RNA was purified according to the supplier´s protocol. The extracted RNA was further purified using QIAamp Viral RNA Mini Kit (QIAGEN) and eluted in 50 μl of ribonuclease-free water. RT-qPCR was performed using the same CDC N1 and N2 probe/primers sets (Integrated DNA Technologies Inc.) which are utilized for COVID-19 diagnostic (https://www.cdc.gov/coronavirus/2019-ncov/lab/rt-pcr-panel-primer-probes.html). RT-qPCR assay targeting pepper mild mottle virus (PMMoV) was performed as viral indicator of human fecal contents and for normalization of SARS-CoV-2 signal (4, 15, 18). Samples were analyzed using GoTaq Probe 1-Step RT-qPCR System (Promega) in 20 μl reaction mix in a QuantStudio 3 Real-Time PCR System (Applied Biosystems). Purified and quantified plasmids containing amplicons from N1 and N2 regions were used to generate the standard curves by serial dilutions. In our conditions, we obtained a standard curve for N1 primer set with an R^2^ of 0.99 with efficiency of 85% (slope= –3.747; y-intercept= 46.076). The N2 primer set generated a standard curve with an R^2^ of 0.98 with an efficiency of 92.3% (slope= –3.508; y-intercept= 42.014). All determinations were made at least in duplicate.

## RESULTS

**FIGURE 2.**
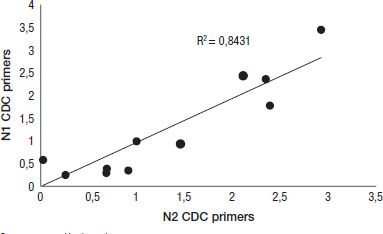
Comparison of SARS-CoV-2 relative RNA concentrations measured in surface water samples using CDC N1 and N2 probe/primers sets

***Source*:** prepared by the authors.

We detected SARS-CoV-2 RNA in all the samples analyzed, and we were also able to quantify the relative SARS-CoV-2 RNA concentration in these samples using both N1 and N2 sets. The RT-qPCR cycle threshold values (Ct) ranged from 32 to 40 with means of 35.4 and 34.4 for N1 and N2, respectively. The comparison of the relative RNA concentrations obtained by the N1 and N2 sets is shown in Figure 2. The correlation showed a R^2^ of 0.84 (Figure 2). In all samples we also determined the relative amount of PMMoV RNA as a viral indicator of human feces content (4, 15), obtaining Ct values ranging from 30 to 33 with a mean of 31.9.

**FIGURE 3.**
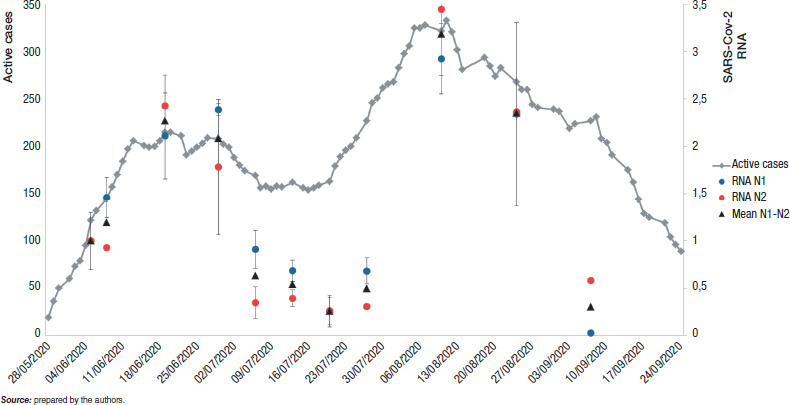
Determinations of relative SARS-CoV-2 RNA concentration and active COVID-19 cases per day. Diamonds (grey) indicate the number of active cases. The relative SARS-CoV-2 RNA for each set of primers is indicated by circles (N1 in blue and N2 in red). The relative RNA mean concentration is represented with triangles. All measurements were made in duplicate, error bars indicate the standard deviation

***Source*:** prepared by the authors.

In order to determine the number of COVID-19 active cases in the Villa Itatí population for each date, we assumed an active case for all positive RT-PCR diagnosis for COVID-19 case beginning from the date of the nasopharyngeal swab collection until the next 14 days, since it is the median period of persistent viral shedding of SARS-CoV-2 in feces and urine (19, 20). As shown in Figure 3, we obtained a curve of active cases for this population based on data provided by the Secretary of Health from the Municipality of Quilmes. We observed an increase in the number of active cases from May 28 until reaching a plateau on June 13 that remained stable until July 1 and then began to decline until July 9, remained stable again until July 23, when a new increase began until reaching the maximum number of active cases on August 11th when the cases began to decline until September 24 coincidentally with the decrease in cases corresponding to the first wave in the community.

The presence of SARS-CoV-2 genetic material was detected at the time when the reported cases in the neighborhood were 122 over 16 478 inhabitants on June 5 (Figure 3), indicating that a sensitivity of less than one reported case per 135 inhabitants could be achieved in this particular setting. These results are summarized in Figure 3 as the mean of the relative RNA concentration for N1 and N2 probe/primers sets. We detected that the change in the SARS-CoV-2 RNA concentration in the water samples obtained for both N1 and N2 matched the trend observed for the number of COVID-19 active cases along time. In other words, the relative SARS-CoV-2 RNA concentration determined in environmental samples traced the shape of infections during the analyzed period.

## DISCUSSION

Our findings showed that as the positive cases increased in the study community, a high concentration of viral RNA in the sewage was observed, while when the positive cases decreased after the outbreak mitigation process and social distancing measures were implemented, lower concentrations of viral RNA were detected in the wastewater samples.

Most studies published to date on the use of environmental surveillance for SARS-CoV-2 have been carried out in high-income settings (3-15). This is the first report of SARS-CoV-2 RNA detection from a surface water source from a low-resource population with precarious wastewater drainage. Additionally, to our knowledge, it is the first experience of utilizing SARS-CoV-2 RNA measurement in wastewater as environmental surveillance strategy for the COVID-19 pandemic in Argentina.

Nevertheless, the study had some limitations, including methodological ones due to the neighborhood infrastructure and the sampling; Determining the hydraulic retention time and sampling more frequently could reduce variation and noise in the data. There were also uncertainties due to limited knowledge about SARS-CoV-2 gastrointestinal infection, the concentration of viral RNA in stool over the course of the illness, the variability in viral dynamics in individuals and fecal shedding (11, 19-22). Because of this, the number of cases could not be estimated with confidence directly from the quantification of viral RNA in wastewater (11).

However, we were able to detect the presence of SARS-CoV-2 genetic material and follow the dynamics of the infection in the community. Our results from this community showed that measurements of SARS-CoV-2 RNA levels in surface water contaminated by sewage can be considered as a reliable estimation of changes in COVID-19 prevalence at the population level. This method may be applied to detect outbreaks or an increase in the number of infected cases and is a useful complement to classic epidemiological surveillance to assess whether the measures applied are effectively working. We believe that the methodology could be improved by increasing the sampling frequency and time of sampling. Although this methodology has its limitations, the trends of SARS-CoV-2 RNA concentration in fecal contaminated surface water may still be useful in supplementing conventional surveillance methods to interpret the trends in community transmission.

A more comprehensive COVID-19 surveillance system could be developed through frequent measurement of SARS-CoV-2 in wastewater, taking into account the disposal, fate, and transport of wastewater in each community. Sewage testing has been successfully used as a method for early detection of other pathogens, such as poliovirus (23). Since SARS-CoV-2 can be shed in the feces of individuals with symptomatic or asymptomatic infection, wastewater surveillance can capture data on both types of infection (19, 21, 22). Thus, it can be a leading indicator of changes in COVID-19 burden in a community and could serve as a COVID-19 indicator that is independent of healthcare-seeking behaviors and access to clinical testing (4, 11).

In conclusion, the detection of SARS-CoV-2 RNA in wastewater can be applied in a low-resource community. In this community, we observed that measurements of SARS-CoV-2 RNA concentrations in surface water contaminated by sewage can be considered to estimate changes in COVID-19 prevalence at the population level.

## Disclaimer.

Authors hold sole responsibility for the views expressed in the manuscript, which may not necessarily reflect the opinion or policy of the *RPSP/PAJPH* and/or PAHO.
